# ZBP1 and heatstroke

**DOI:** 10.3389/fimmu.2023.1091766

**Published:** 2023-02-10

**Authors:** Fanglin Li, Jiayi Deng, Qiuli He, Yanjun Zhong

**Affiliations:** ^1^ Critical Care Medicine, The Second Xiangya Hospital, Central South University, Changsha, China; ^2^ Department of Critical Care Medicine and Hematology, The 3rd Xiangya Hospital, Central South University, Changsha, China; ^3^ Department of Nephrology, The First Affiliated Hospital of Gannan Medical University, Ganzhou, China

**Keywords:** ZBP1, heatstroke, RIPK3, programmed cell death, necroptosis

## Abstract

Heatstroke, which is associated with circulatory failure and multiple organ dysfunction, is a heat stress-induced life-threatening condition characterized by a raised core body temperature and central nervous system dysfunction. As global warming continues to worsen, heatstroke is expected to become the leading cause of death globally. Despite the severity of this condition, the detailed mechanisms that underlie the pathogenesis of heatstroke still remain largely unknown. Z-DNA-binding protein 1 (ZBP1), also referred to as DNA-dependent activator of IFN-regulatory factors (DAI) and DLM-1, was initially identified as a tumor-associated and interferon (IFN)-inducible protein, but has recently been reported to be a Z-nucleic acid sensor that regulates cell death and inflammation; however, its biological function is not yet fully understood. In the present study, a brief review of the main regulators is presented, in which the Z-nucleic acid sensor ZBP1 was identified to be a significant factor in regulating the pathological characteristics of heatstroke through ZBP1-dependent signaling. Thus, the lethal mechanism of heatstroke is revealed, in addition to a second function of ZBP1 other than as a nucleic acid sensor.

## 1 Introduction

Heatstroke is generally regarded as one of the most dangerous illnesses, with rapid progression after onset. The primary feature of heatstroke is a high core body temperature, which is caused by strenuous exercise or exposure to hot environments, and the direct cause is the imbalance of heat metabolism, manifested as the heat generation being greater than the heat dissipation (1). Clinically, heatstroke is defined as extreme hyperthermia (usually over 40.0°C), central nervous system (CNS) dysfunction, and multiple organ dysfunction multiple organ dysfunction syndrome (MODS), which are caused by the complex interaction between thermal-related cytotoxicity, inflammatory response, and coagulation abnormalities (2–4). Depending on the trigger, heatstroke is clinically classified as either classic heatstroke (CHS) or exertional heatstroke (EHS). The direct cause of both types is high temperature; however, despite their clinical manifestations being similar, the mechanisms of the two slightly differ. CHS is mainly caused by passive exposure to high temperatures, while EHS is mainly caused by strenuous exercise. The former involves the absorption of too much heat from the environment, while the latter involves the production of too much endogenous heat due to an accelerated metabolism (3). As for the survival rate of heatstroke, the reported mortality rate was as high as 23.3%, and the death rate from heatstroke has been predicted to increase as a result of climate change and the estimated whole world increase in the frequency and intensity of heat waves (5–8). Although adequate lowering of the body temperature and timely treatment can be administered, heatstroke remains hazardous, and individuals who do survive from heatstroke may suffer long-lasting neurological sequelae (7, 9).

**Figure 1:**
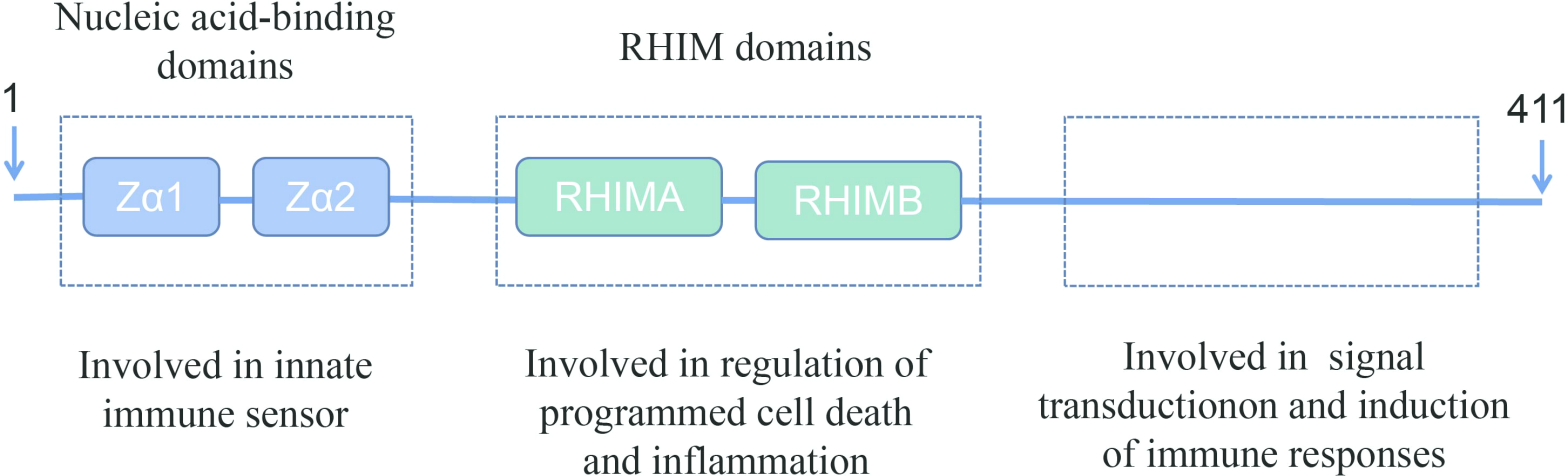
Schematic representation of full-length ZBP1. ZBP1 encodes two N-terminal Z-DNA-binding domains, which are reported to bind to Z-DNA, B-DNA, and RNA. ZBP1 also has two receptor-interacting protein homotypic interaction motif (RHIM) domains in the central part that facilitate the interaction with other RHIM domain-containing proteins. These RHIM domains are important in mediating ZBP1-dependent cell death and inflammatory responses (13, 14).

Z-DNA-binding protein 1 (ZBP1) was originally identified as an interferon (IFN)-inducible tumor-associated protein involved in the host response to tumors (10). The amino terminus of ZBP1 contains two nucleic acid-binding domains and two receptor-interacting protein homotypic interaction motif (RHIM) domains (11, 12). The nucleic acid-binding domains sense and bind viral nucleic acids and are involved in the innate immune response. One the other hand, the RHIM domains bind to homologous proteins and are involved in the regulation of cell death and inflammation. The carboxy-terminal of ZBP1 has been reported to be involved in signal transduction (**Figure 1**) (12, 15, 16).

**Figure 2:**
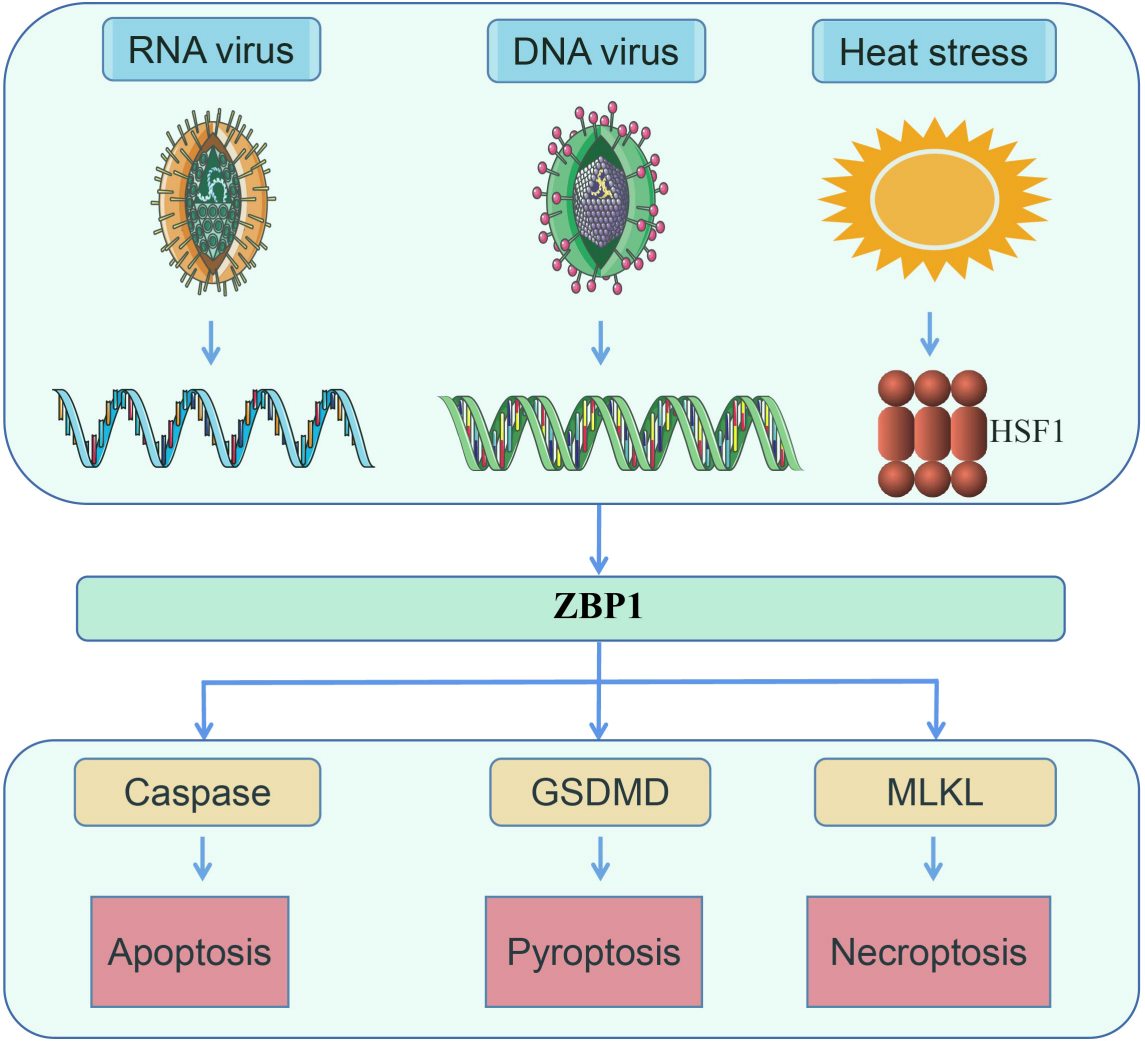
ZBP1 senses RNA virus infection, DNA virus infection, and heat stress, following which the necrotic complex will be assembled to induce apoptosis, pyroptosis, and necroptosis (14, 16).

Numerous studies on heatstroke and ZBP1 have been published, including several review articles. Despite such research, at present, there are no reports on the relationship between heatstroke and ZBP1 (1–3, 7, 17–19). Studies on heatstroke over the past several decades have suggested that heatstroke results from the complex interaction of thermoregulatory failure with the amplification of acute-phase response (3, 4, 7, 9, 20). In a recent study by us, a novel function of ZBP1 in mediating heatstroke has been identified, which not only supplemented the understanding of the pathogenesis of heatstroke but also revealed a second function of ZBP1 other than as a nucleic acid sensor (**Figure 2**) (21). In the present review, the latest research advances on ZBP1 are summarized, as well as the pathogenesis and pathophysiology of heatstroke.

## 2 The Role of ZBP1

### 2.1 ZBP1 in viruses

#### 2.1.1 ZBP1 in influenza A virus

Influenza A virus (IAV) is a negative-sense RNA virus that is cytotoxic to most cell types in which it replicates. The primary IAV host is aquatic birds, with virus replications occurring in the gastrointestinal tract that commonly are asymptomatic. However, mammalian IAV strains replicate in respiratory tissues and produce symptoms that range from mild cases to severe and sometimes fatal (13). Owing to the high mutation rates and the genetic recombination thereof, IAV is commonly associated with epidemics (22). Several pattern recognition receptors (PRRs), including Toll-like receptors (TLRs), RIG-I-like receptors (RLRs), and nucleotide-binding domain leucine-rich repeat-containing receptors (NLRs), have been found to be involved in IAV infection (22). Virus recognition by these receptors triggers an innate immune response to restrict virus replication and clear the infection. The sensing of IAV initiates a variety of intracellular signaling pathways.

In a previous study, ZBP1 was found to be able to sense nucleoprotein (NP) and polymerase subunit PB1, which are components of IAV, and to promote activation of the nucleotide-binding domain leucine-rich repeat and pyrin domain-containing receptor 3 (NLRP3) inflammasome and pro-inflammatory cytokine production *via* receptor-interacting protein kinase 3 (RIPK3) signaling. At the same time, ZBP1 is regulated by IFN regulatory factor (IRF1) (23, 24). Aside from caspase-8, caspase-6 has also been reported to promote ZBP1-mediated programmed cell death (PCD) during IAV infection. The authors also found that pyroptosis, which is another PCD, could mediate cell death during IAV infection (25). In addition, regarding selectively deficient apoptosis in an IAV infection mouse model, necroptosis could completely compensate for the role of apoptosis (26). In further research, ZBP1 was also discovered to be able to sense IAV genomic RNA, bind to RIPK3, and trigger both the necroptosis and apoptosis of PCD to clear away infected cells and accordingly protect the host (27). In addition, a new pathogen-associated molecular pattern, Z-RNA, generated from replicating IAV, could activate ZBP1 *via* the Zα2 domain thereof in infected cells (28, 29). Despite these findings on the mechanisms promoting ZBP1 activation to induce PCD during IAV infection, further studies have confirmed the involvement of viral sensing of the IAV viral ribonucleoprotein (vrnP) complexes and sensing of the IAV RNA by retinoic acid-inducible gene I (RIG-I), which then triggered ZBP1-related cell death (30). Moreover, IAV can also induce the ubiquitination of ZBP1, and tripartite motif 34 (TRIM34) has been notably reported to be involved in regulating IAV-induced PCD by mediating the K63-linked polyubiquitination of ZBP1 (24, 30, 31). Thus, to a certain extent, these findings together explain the activation of ZBP1 during infection with IAV.

#### 2.1.2 ZBP1 in cytomegalovirus

Cytomegaloviruses (CMVs), which belong to the herpesvirus family, are enveloped double-stranded DNA (dsDNA) viruses with a genome size of approximately 235 kbp. CMVs are slowly replicated in several specific cell types (32). The CMV genomes include over 200 independent genes with the potential to encode antigenic proteins. They also contain a common core of genes conserved in all herpesviruses, which is in the central 100 kbp. These gene families encode most of the glycoproteins that are required for virus replication (32).

As early as 2009, protein 45 of the mouse CMV (M45) was identified as a RHIM-containing protein that could bind to ZBP1 *via* the functional RHIMs thereof when overexpressed. At the same time, M45 can inhibit the phosphorylation of RIP3 and has the potential to disrupt ZBP1–RIP interactions, suggesting that it is significant in the suppression of cell death during mouse CMV (MCMV) infection (33). Notably, M45 was found to promote self-assembly into amyloid fibrils and interact with the RHIM-containing domain so as to form heteromeric amyloid fibrils and mediate necroptosis (34). Further studies verified that the MCMV-encoded viral inhibitor of RIP activation (vIRA) serves a similar function to M45, i.e., the targeting of ZBP1–RIP3 complexes, as well as the suppression of PCD induced by these complexes (35, 36). Recently, viral immediate-early protein 3 (IE3)-dependent transcription has been found to be indispensable for ZBP1-mediated cell death, and both the Zα2 and RHIM domains are required for MCMV-mediated necroptosis (37). Human cytomegalovirus (HCMV), much like MCMV, can also interact with ZBP1, which triggers strong antiviral immune responses (14, 38, 39).

#### 2.1.3 ZBP1 in herpes simplex virus 1

Herpes simplex virus 1 (HSV-1) belongs to the alphaherpesvirinae subfamily. It is a dsDNA virus with a genome size of approximately 152 kbp. To date, over 80 genes encoded by HSV-1 have been confirmed (40). HSV-1 primarily infects epithelial cells, manifests vesicular eruptions (mainly in the oral or genital mucosa), and has lifelong latency in neurons (41, 42). Primary infection of HSV-1 generally occurs at a young age, with the great majority of infection causing mild clinical symptoms in immunocompetent people, in addition to the fairly high mortality rate in neonates (43). Approximately 3.7 billion individuals aged under 50 years are infected with HSV-1 worldwide (44).

In an earlier study in 2012, ZBP1 was found not necessary for HSV-1 DNA sensing in HepG2 hepatocellular carcinoma cells. However, ectopic ZBP1 expression repressing the HSV-1 viral replication depends on the Zβ and D3 domains rather than the Zα domain (45). In further research, HSV-1 ICP6 was identified as a RHIM-containing viral inhibitor analogous to vIRA/M45, which can target RIPK3 and function as a ZBP1–RIPK3 signaling inhibitor, thereby inducing death in murine cells and survival in human cells (46). ICP6 was also confirmed to promote tumor necrosis factor (TNF) receptor 1-induced necrosome assembly (47). Notably, in astrocytes, HSV-1 can also induce necroptosis through ZBP1 signaling (48). In a study on HSV-2-infected primary vaginal epithelial cells, it was found that ZBP1 was also required to recognize HSV-2 (49). In a recent study, it has been revealed that ZBP1 can interact with AIM2 and pyrin inflammasome components and mediate multiple pathways of PCD, including apoptosis, necroptosis, and pyroptosis (50).

#### 2.1.4 ZBP1 in other viruses

In addition to IAV, CMV, and HSV, a variety of diverse viruses, including vaccinia virus (VV) (51–55), Zika virus (ZIKV) (56–58), West Nile virus (WNV) (57), severe acute respiratory syndrome coronavirus 2 (SARS-CoV-2) (59–61), Japanese encephalitis virus (JEV) (62), and human immunodeficiency virus type 1 (HIV-1) (63), have been demonstrated to induce or repress ZBP1-mediated PCD during infection. Together, these studies clearly illustrate that ZBP1-mediated PCD acts as a significant defense mechanism against viral infection.

#### 2.1.5 ZBP1 in other pathogenic microorganisms

ZBP1 is not only involved in virus-mediated PCD, but it has also been reported in other pathogenic microorganisms such as bacteria (64–67), fungi (50, 68), and parasites (69, 70). Similar to its function in viruses, ZBP1 promotes the induced inflammasome activation and PCD of these microorganisms, but the mechanisms of the natural ligands or signals that activate the inflammasome and PCD remain unclear.

### 2.2 ZBP1 in tumors

ZBP1 was initially identified in the tumor stroma and tumor cells as an IFN-inducible protein that senses Z-form nucleic acids, but was later confirmed in the cytosolic sensing of both DNA and RNA (10, 11, 28). Recently, ZBP1 has been found to be involved in the occurrence and development of various tumors, including breast cancer (71–74), melanoma (75–77), colorectal cancer (76, 77), non-small cell lung cancer (76), multiple myeloma (78), ovarian cancer (79), urothelial carcinoma (80), fibrosarcoma (71), and kidney renal clear cell carcinoma (81). A common feature of these studies is the finding that ZBP1 mediates PCD in the aforementioned tumors. However, several of these aforementioned studies were performed in cell lines and there was a lack of animal experiments. Moreover, the specific mechanism of ZBP1-mediated PCD in tumor cells remains unclear. As such, further studies including animal experiments are still required.

### 2.3 ZBP1 in adenosine deaminase acting on RNA 1

More recently, studies have revealed that RNA-specific adenosine deaminase 1 (ADAR1) is involved in ZBP1-mediated signaling. ADAR1 belongs to the adenosine deaminase acting on RNA (ADAR) family. ADARs were initially found to have dsRNA unfolding activities (82), but were later confirmed to be adenosine-to-inosine RNA editing enzymes (83). ADAR1 has a catalytic deaminase domain and three dsRNA-binding domains, with two different functional isoforms: ADAR1p110 and ADAR1p150. The p110 isoform contains one Z-DNA-binding domain, Zβ, while the p150 isoform not only has the Zβ but also the Zα domain (84). ADAR1 also has editing-dependent functions, can regulate innate immunity, and alters miRNA maturation (85).

A breakthrough was recently made with regard to the research on ADAR1 and ZBP1. In a previous study using murine colorectal cancer and melanoma models, ADAR1 was found to suppress ZBP1-mediated inflammasome activation and PCD, including apoptosis, necroptosis, and pyroptosis (77). Most recently, several Nature articles have reported that ADAR1 could inhibit Z-RNA accumulation and ZBP1-dependent cell death by preventing the accumulation of mRNA transcripts. In parallel, further studies have identified ADAR1 as a negative regulator of sterile ZBP1 activation by means of ADAR1 mutations (75, 86). Through ADAR1 mutations, ZBP1 could also contribute to type I interferonopathies (87). With further in-depth research, the Zα domain of ADAR1 was confirmed to interact with ZBP1 and inhibit its signaling (88).

### 2.4 ZBP1 in heatstroke

The molecular mechanism of heatstroke pathogenesis remains unclear. The focus of previous research has been on the nucleic acid receptor function of ZBP1 and its mediation of PCD. In our recent studies, ZBP1 has been found to cause heatstroke pathology by activating the RIPK3-induced activation of mixed lineage kinase domain-like pseudokinase (MLKL)-dependent necroptosis and caspase-8-dependent cell death both *in vivo* in genetic mouse models and *in vitro* in cultured cells (21). A further study found that heat stress increased the expression of ZBP1 through HSF1 and promoted the aggregation of ZBP1 fusion proteins, which was followed by the recruitment of RIPK3, phosphorylation of MLKL, and the cleavage of caspase-8 (21).

## 3 Pathogenesis and pathophysiology of heatstroke

**Figure 3:**
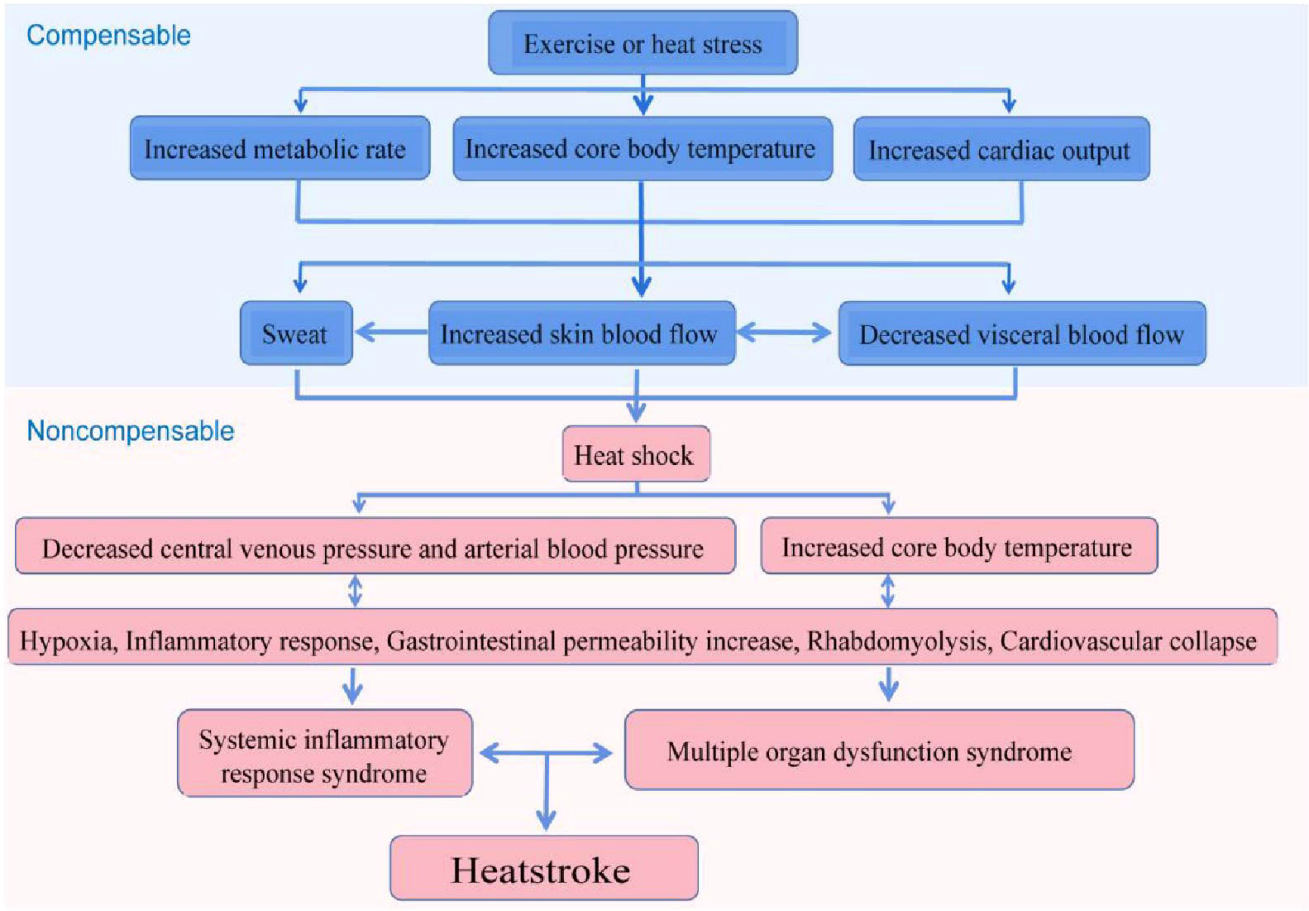
Possible pathophysiological pathway leading to heatstroke. The sequence of events leading to heatstroke involves a transition from a compensable thermoregulatory state to the non-compensable condition. Heat stress initiates a thermoregulatory response to maintain the balance of heat production and heat dissipation. When the arterial blood pressure begins to decrease substantially, the core temperature begins to increase rapidly and becomes non-compensable. This thermoregulatory failure aggravates pathophysiological processes, including the inflammatory response and multi-organ dysfunction, and is ultimately expressed as heatstroke (3).

In the early phase of heatstroke, the organism is in a status of compensation, in which a balance is maintained between heat dissipation and heat generation. Upon entering the late stage of heatstroke, the organism produces more heat than it dissipates, with the cardiac output being reduced and is becoming inadequate to deal with the high thermoregulatory demands, ultimately leading to a status of decompensation. The heatstroke injury to the organism mainly occurs in the decompensation stage, which results in thermal-related cytotoxicity, inflammatory responses, and coagulation responses, eventually causing MODS, disseminated intravascular coagulation (DIC), and even death (**Figure 3**) (1–3, 89, 90). Such pathological features of heatstroke were found to be mediated by ZBP1 through the triggering of the RIPK3-induced activation of MLKL-dependent necroptosis and caspase-8-dependent cell death, which was identified *in vivo* in genetic mouse models and *in vitro* in cultured cells (21).

### 3.1 Hypoxia and oxidative stress

Upon entering the non-compensable phase, the cardiac output is reduced and becomes inadequate to deal with the high thermoregulatory demands; subsequently, hypoxia occurs. Hypoxia further induces the generation of reactive oxygen species (ROS) (91–101). Several possible mechanisms cause the production of ROS during heatstroke. Li et al. (91) found that heat stress can promote the activation of the p38–MK2 signaling pathway, which promotes apoptosis by regulating the accumulation of ROS. Heat stress can also stimulate the production of ROS by inducing mitochondrial fission or inhibiting the activity of mitochondrial complex I (92, 93). Kikusato and Toyomizu (94) showed that the change in the mitochondrial membrane potential may be a significant factor in the overproduction of ROS under heat stress stimulation. Wang et al. (101) reported that heat stress can induce the production of ROS *via* the upregulation of Ang II and the receptor AT1 thereof.

The generated ROS can oxidatively damage lipids, proteins, and DNA, among others. The mitochondria represent the main production site of ROS and also its main active site, with damage to the mitochondria being able to induce apoptosis (91–95, 97, 102, 103). Under heat stress, the generated ROS can induce necroptosis and pyroptosis (103–105). Studies have revealed that ROS can induce apoptosis by promoting the release of apoptotic factors (105). Several studies have also demonstrated that ROS are pivotal upstream factors in the progress of apoptosis induced by heat stress (103, 106, 107). They can promote heat stress-induced apoptosis *via* ERK and Bcl-2 signaling (103). The accumulation of ROS, in particular O_2_^−^, promotes the elevation of Ca^2+^ in the cytoplasm *via* upregulating the inositol triphosphate receptor (IP3R), and the increased Ca^2+^ then induces apoptosis (108). Notably, similar results were found in another study, wherein heat stress induced apoptosis through the ROS-, Ca^2+^-, and p53-dependent translocation of Bax (106). Apoptosis can also be prevented by scavenging ROS (107, 109). In a recent study by us, heat stress has been found to be able to induce necroptosis, apoptosis, and pyroptosis *via* the ZBP1–RIPK3 signaling pathway, genetic deletion, or the targeted inhibition of ZBP1, RIPK3, or both caspase-8 and MLKL, which can prevent the multiple forms of cell death and improve survival. Although ROS was not directly detected, heat stress induced the generation of intravascular thrombin, and the occlusion of the microcirculation was attenuated by ZBP1 and RIPK3 deficiency (21). Therefore, a reasonable assumption could be made that blocking this pathway could reduce the production of ROS, then mitigating cell death. However, these findings require further validation.

### 3.2 Inflammatory response

Hyperthermia syndrome is an ongoing exacerbation process that consequently leads not only to a direct heat-related cytotoxicity effect but also to an inflammatory response. The heat stress-induced inflammatory response is considerably similar to that in systemic inflammatory response syndrome (SIRS) (2, 3). As is well known, SIRS can lead to rapid deterioration of the clinical status, resulting in multi-organ dysfunction, DIC, and death (110). Hence, akin to SIRS, heatstroke is considered a heat-related SIRS (2, 3). In a clinical study including patients with heatstroke, approximately 84% at the same time met the diagnostic criteria for SIRS (111). Existing studies have suggested that cytokines are involved in the heatstroke responses from patients with heatstroke to experimental animal models. Both clinical and experimental pieces of evidence have revealed multiple cytokines elevated in plasma and tissues, including both pro-inflammatory [such as interleukin-1 beta (IL-1β) and tumor necrosis factor alpha (TNF-α)] and anti-inflammatory [such as interleukin-6 (IL-6) and interleukin-10 (IL-10)] cytokines (3, 112). As such, multi-organ dysfunction is considered to be caused by heat-related cytotoxicity combined with the subsequent SIRS (2).

Cytokines are immune modulators that are released by a variety of cells, including immune and non-immune cells, e.g., macrophages, endothelial cells, T cells, B cells, and microglia, which act as intercellular chemical messengers (113). Elevated levels of cytokines can be detected in the plasma of patients and experimental animal models. These elevated cytokines include both pro-inflammatory [e.g., IL-1β, TNF-α, and interferon gamma (IFN-γ)] and anti-inflammatory (e.g., IL-6 and IL-10) cytokines (2, 3, 7, 112, 114). The plasma levels of cytokines differ during the progress of heatstroke, and loss of the balance between pro- and anti-inflammatory cytokines may result in inflammation-associated injury and immunosuppression. Previous studies in humans, rats, and mice have confirmed that heatstroke promotes systemic and local generation of pro-inflammatory cytokines (i.e., IL-1β, IL-6, IFN-γ, and TNF-α) (115–118). The levels of TNF receptors and IL-6 have been reported to be correlated with the severity of heatstroke (119, 120).

Studies conducted on the regulation of the inflammatory response of heatstroke in rat models revealed that neutralizing the inflammatory mediators with recombinant activated protein C, cytokine receptor antagonist, or corticosteroids reduced tissue injury, resulting to an improved survival (121, 122). However, increased mortality was observed in studies involving baboons that received corticosteroids and involving mice, in which the expression levels of the TNF-α receptor and IL-6 were deficient (114, 123). Consistent with prior research, a previous study by us that used a mouse model also showed elevated serum levels of pro-inflammatory cytokines (i.e., IL-1β, IL-6, TNF-α, and IL-1α) and the genetic deletion of RIPK3 or ZBP1 in mice, which significantly reduced the serum levels of the aforementioned pro-inflammatory cytokines (21). The described evidence supports the inflammatory response possibly being either beneficial or detrimental, depending on the phase and magnitude of the induced response.

### 3.3 Coagulopathy

Coagulation disorders are prominent features in patients with heatstroke and in animal models. Heatstroke has been identified to be associated with the activation of coagulation, anti-coagulation, and fibrinolytic systems (124–132). There are several different components of the coagulation, anti-coagulation, and fibrinolytic pathways that have been identified to be altered during heatstroke both in human and animal models, such as increased plasminogen activator inhibitor 1 (PAI-1), increased thrombin/anti-thrombin (TAT) complex, thrombocytopenia, decreased fibrinogen (Fib) levels, increased D-dimer levels, delayed prothrombin time (PT), and delayed activated partial thromboplastin time (APTT) (21, 133, 134). The most serious coagulopathy complicated by heatstroke is DIC, and the mortality rate of patients with DIC is significantly increased. Pathological manifestations such as congestion, hemorrhage, endothelial cell injury, extensive microthrombosis, and necrosis were observed in multiple organs in heatstroke rats (135). In our study recently published in Science, thrombin activation, fibrin deposition, and platelet adhesion were directly observed in a mouse model of heat stress using spinning disk confocal intravital microscopy (SD-IVM) (21). Furthermore, genetic deletion of ZBP1 or RIPK3 obviously decreased the thrombin activation, fibrin deposition, and platelet adhesion in the liver microvasculature of heat-stressed mice, while the plasma levels of the anticoagulant components PAI-1 and TAT complex were significantly decreased (21).

During the pathogenesis of heatstroke, various factors such as high temperature, ischemia, hypoxia, and inflammatory responses can lead to disorders of the coagulation system and even induce DIC. Clinically, DIC is characterized by extensive microthrombosis and bleeding due to damage to the microvascular system, activation of coagulation, excessive consumption of coagulation factors, and secondary hyperfibrinolysis (2). It is a fatal complication of heatstroke that can initiate during the recovery period and is thought to be a significant mechanism of fatality (3, 7, 129). The endothelium regulates vascular permeability and leukocyte movement, and it maintains the balance between procoagulants, anticoagulants, and fibrinolysis. Previous *in vitro* studies have revealed that heat stress induces a hypercoagulable state, increases vascular permeability, and promotes the expression of adhesion molecules (3, 7, 129). *In vitro* studies have also suggested that heat directly activates platelets, promotes platelet aggregation, and causes hyper-aggregation (129, 136, 137). Hyperthermia also results in the increased concentrations of platelet-derived microparticles (PMPs) and endothelial-derived microparticles (EMPs) in the circulation (138). EMPs have been reported to be involved in promoting endothelial oxidative stress, diminishing nitric oxide bioavailability, enhancing cell adhesion molecule expression, and impairing the endothelial vasomotor function (138, 139). PMPs, which are released as a result of platelet activation, cause endothelial injury and contribute to a hypercoagulable state (138–140). Circulating levels of several platelet aggregation-promoting components [such as von Willebrand factor (vWF) and thrombomodulin (TM)] and intercellular adhesion molecules are elevated, which indicates an interaction between endothelial cells and leukocytes *in vivo* (128, 141).

Tissue factor (TF) is the initiator of the coagulation process and is widely expressed in various tissues and cells. It initiates the coagulation cascade *via* the extrinsic coagulation pathway (142). Bouchama et al. reported that, in a baboon model of heatstroke, inhibiting TF with recombinant anticoagulant protein significantly attenuated the coagulation activation. However, neutralizing TF had no apparent effect on fibrinolysis and inflammation. These findings suggest that TF is the main trigger of coagulopathy under such conditions, but it is dispensable for tissue damage and organ dysfunction (143). At present, the specific role of TF on coagulation during heat stress remains unclear, and further research is still needed.

### 3.4 Tissue injury responses

Heat stress induces extensive tissue damage, including damage to the liver, lung, kidney, spleen, gastrointestinal tract, brain, and skeletal muscles. Although these injuries are commonly seen in human and animal studies (3, 4, 7, 21, 115), most of the tissue damage and organ dysfunction are attenuated after the genetic deletion of ZBP1 or Ripk3, as shown in our previous study (21). A typical symptom of heatstroke is CNS dysfunction, which manifests as varying degrees of mental status changes (8, 112, 144, 145). An elevated brain temperature is caused by the combination of an increased cerebral metabolic rate and a decreased cerebral blood flow (112). A decrease in cerebral blood flow causes cerebral ischemia, hypoxia, cerebral edema, the generation of ROS, activation of the microglia, disruption of the blood–brain barrier, an increase in the permeability of the blood–brain barrier, and further promotion of the leakage of vascular contents into the brain. The aforementioned series of responses promote the occurrence of neuroinflammation and neuronal damage. Neuroinflammation and neuronal damage may directly cause cognitive dysfunction (74, 115, 116, 118, 119, 146, 147). As reported in previous studies, 100% of patients with heatstroke presented acute neurological symptoms, with a total of 71.4% of the impaired patients developing long-term cerebellar dysfunction and approximately 34.4% of the survivors being left with long-lasting neurological damage, which may be associated with irreversible brain damage (8). However, the role of the brain in the progression of heatstroke remains unknown. Further research on the pathogenesis of encephalopathy is still needed.

Extensive peripheral tissue damage has been found in both patients and animals with heatstroke, and the commonly used index to assess the damage to peripheral tissues and organs is the alteration in the serum enzyme levels. For instance, elevation of the levels of creatine kinase, creatine kinase-MB, and alanine aminotransferase can indicate damage to different organs (3, 4). Histopathology has also fully confirmed the morphological and other pathological changes in patients and animals with heatstroke (4, 148–150). When heat stress persists, the blood flow is redistributed, which is manifested as a decreased visceral blood flow and an increased blood flow to the skin and vital organs, so as to meet the needs of heat dissipation. These factors lead to intestinal ischemia, which induces the generation of intestinal free radicals and intestinal mucosal edema and increases intestinal permeability, all of which promote intestinal disruption and lead to the release of a large number of intestinal toxins into the blood circulation (13, 109, 151–158). Renal failure is a common complication of heatstroke that is mainly manifested as tubular necrotizing acute renal failure and can be caused by massive protein deposition in the renal tubular epithelial cells after hemolysis and rhabdomyolysis (13, 21, 158–166). Liver dysfunction in patients with heatstroke and in animal models is considerably common, with the liver injury mainly occurring in the recovery period of heatstroke (167–174). Under heat stress, the liver can actively release various inflammatory mediators such as high-mobility group box-1 (HMGB1), which aggravates liver damage, suggesting that this factor may be a consequence of the inflammatory response that ensues during recovery rather than an acute response to heat stress (21, 97, 104, 112, 175). Liver injury under heat stress has also been considered to be associated with a large proportion of hepatocyte cell death, functional disorders of Kupffer cells, and abnormal expression of heat-related proteins, among other factors (21, 174). During the heatstroke process, the lungs are the most vulnerable and injured organs owing to their unique structure. Patients mainly present with acute lung injury (ALI), accompanied by pulmonary edema, pulmonary hemorrhage, pleural effusion, and exudation of inflammatory cells and cytokines (150, 176, 177). ALI was also found in mouse and rat models of heatstroke (21, 99, 158, 177–183). Rhabdomyolysis is more common in patients with EHS, and several possible mechanisms cause such phenomenon (3, 166, 184–193). One potential mechanism is the elevated muscle tension caused by strenuous activity, which increases the chance of tissue injury (184). Direct thermal cytotoxicity of hyperthermia may be another cause. Alterations of the calcium channels in muscle cell membranes have also been reported to be involved in rhabdomyolysis (7, 194–196). In parallel, more detailed studies revealed that heat stress-induced rhabdomyolysis could be partly attributed to the Ca^2+^-dependent increases in the levels of reactive oxygen and nitrogen, which exacerbated muscle membrane injury (7, 197). Another potential mechanism of rhabdomyolysis is dehydration, which increases blood redistribution and reduces the skeletal muscle blood flow. Such changes make the skeletal muscles ischemic, cause hypoxia, and increase Ca^2+^, which in turn increases the activation of the proteolytic enzymes in intramuscular Ca^2+^ that contribute to skeletal muscle breakdown, eventually causing rhabdomyolysis (198, 199). Ferroptosis has also been reported to mediate rhabdomyolysis (193).

### 3.5 Gastrointestinal integrity and flora imbalance

During heat stress, the body’s blood flow is redistributed in order to maintain the balance between heat generation and heat dissipation. This redistribution results in a decrease in splanchnic blood flow, causing gastrointestinal ischemia and hypoxia, and the generation of ROS and nitrogen species. Furthermore, the damage to the intestinal mucosa and cells is in turn aggravated and the permeability of the intestinal mucosal barrier increased, thereby allowing toxins and pathogens to leak into circulation, which further aggravates multiple organ injury (200). These factors also result in the gastrointestinal flora imbalance, including the decreased diversity and abundance of flora, which is mainly manifested as a decrease in beneficial bacteria and an increase in pathogenic bacteria (201, 202). In turn, the increase in pathogenic bacteria promotes intestinal inflammation and disrupts the intestinal barrier, resulting in a vicious cycle (203). The results of our previous study using a heatstroke mouse model also revealed the loss of intestinal integrity and inflammatory cell infiltration (21).

## 4 Conclusions

**Figure 4:**
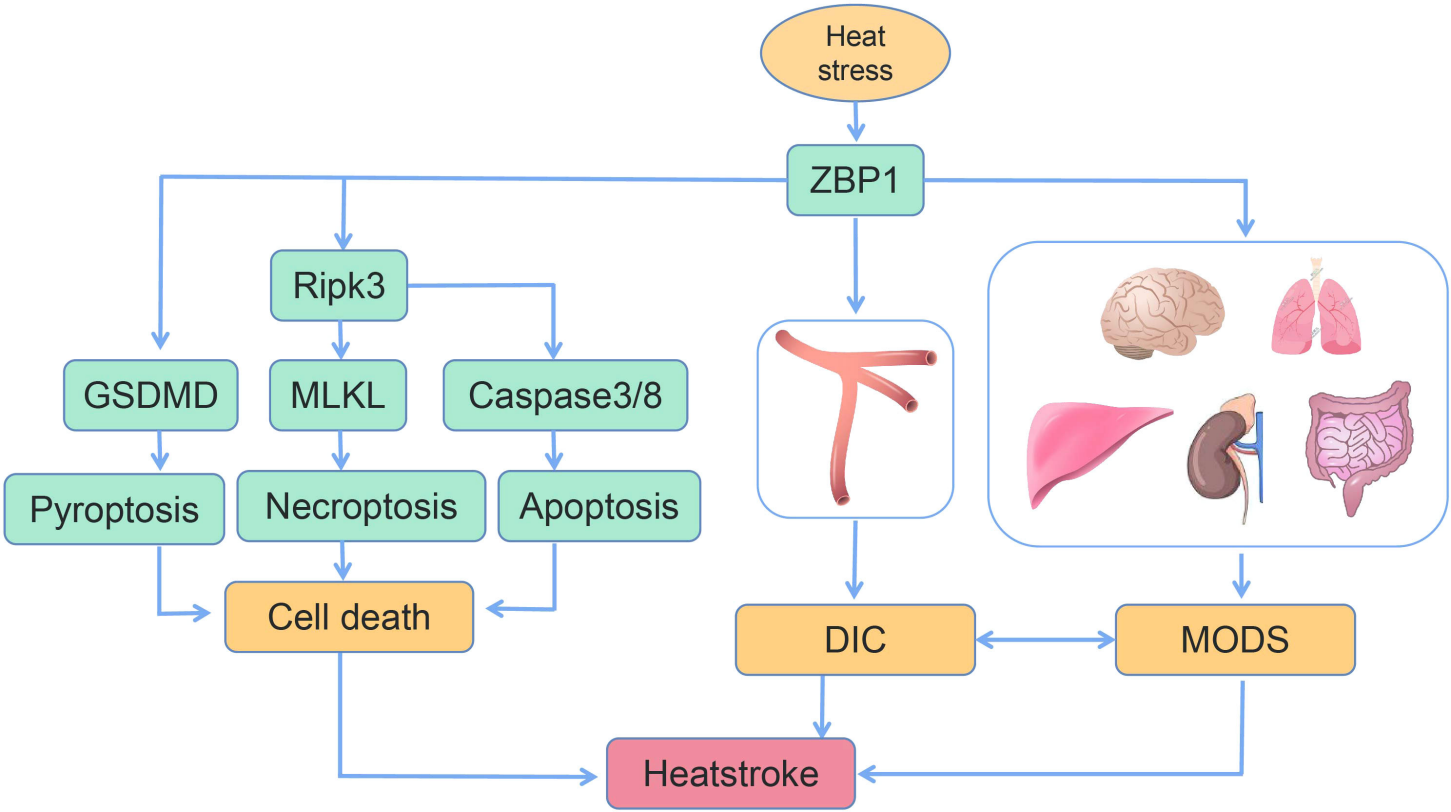
Schematic of the mechanism of heat stress-induced programmed cell death, disseminated intravascular coagulation (DIC), and multiple organ dysfunction syndrome (MODS) (14, 16).

In the present review, the most recent advances with regard to ZBP1 and the pathogenesis of heatstroke were summarized. Recent research has indicated that the clinical characteristics of heatstroke result from the complex interaction between thermal-related cytotoxicity, inflammatory response, coagulopathy, rhabdomyolysis, and gastrointestinal disruption. In a most recent study by our group, in addition to demonstrating the features of heatstroke in a mouse model, it was also confirmed that genetic deletion or targeted inhibition of the ZBP1 pathway, this pathway being a significant factor in heatstroke, was able to significantly improve organ function, reduce multiple organ injury, inhibit DIC, and, most notably, improve survival (**Figure 4**). Another function of ZBP1, in addition to being a nucleic acid sensor, was also discovered. These findings are of considerable significance in revealing the pathogenic mechanism of heatstroke. However, the present understanding of the pathogenesis and pathophysiology of heatstroke is still incomplete. Additional research is still needed to fully elucidate the pathogenic mechanism of heatstroke so as to provide critical direction and basis for its clinical diagnosis and treatment, thereby reducing morbidity and improving patient survival.

## Author contributions

FL, QH, and YZ wrote the main manuscript text. JD prepared **Figures 1**–**4**. All authors reviewed the manuscript. All authors contributed to the article and approved the submitted version.

## Glossary

**Table:**

ZBP1	Z-DNA-binding protein 1
DAI	DNA-dependent activator of IFN-regulatory factors
CNS	central nervous system
MODS	multiple organ dysfunction syndrome
CHS	classic heatstroke
EHS	exertional heatstroke
RHIM	receptor-interacting protein homotypic interaction motif
IAV	influenza A virus
PRRs	pattern recognition receptors
TLRs	Toll-like receptors
RLRs	RIG-I-like receptors
NLRs	nucleotidebinding domain leucine-rich repeat-containing receptors
NP	nucleoprotein
NLRP3	nucleotide-binding domain leucine-rich repeat and pyrin domain-containing receptor 3
RIPK3	receptor-interacting protein kinase 3
IRF1	IFN regulatory factor
PCD	programmed cell death
RIG-I	retinoic acid-inducible gene I
TRIM34	tripartite motif 34
CMV	cytomegalovirus
M45	protein 45 of the mouse CMV
MCMV	mouse CMV
vIRA	viral inhibitor of RIP activation
IE3	immediate-early protein 3
HCMV	human cytomegalovirus;
dsDNA	double-stranded DNA
VV	vaccinia virus
ZIKV	Zika virus
WNV	West Nile virus
SARS-CoV-2	severe acute respiratory syndrome coronavirus 2
JEV	Japanese encephalitis virus
HIV-1	human immunodeficiency virus type 1
HSV1	herpes simplex virus 1
AIM2	absent in melanoma 2
ADAR1	adenosine deaminase acting on RNA
DIC	disseminated intravascular coagulation
ROS	reactive oxygen species
IP3R	inositol triphosphate receptor
SIRS	systemic inflammatory response syndrome
IL-1b	interleukin-1 beta
TNF-a	tumor necrosis factor alpha
IL-6	interleukin-6
IL-10	interleukin-10
IFN-g	interferon gamma
PAI-1	plasminogen activator inhibitor 1
TAT	thrombin–anti-thrombin
Fib	fibrinogen
PT	prothrombin time
APTT	activated partial thromboplast
SD-IVM	spinning disk confocal intravital microscopy
PMPs	platelet-derived microparticles
EMPs	endothelial-derived microparticles
vWF	von Willebrand factor
TM	thrombomodulin
TF	tissue factor
ALI	acute lung injury
GI	gastrointestinal
HMGB1	high-mobility group box-1
